# Key stakeholders’ experiences of respite services for people with dementia and their perspectives on respite service development: a qualitative systematic review

**DOI:** 10.1186/s12877-017-0676-0

**Published:** 2017-12-07

**Authors:** Emma O’ Shea, Suzanne Timmons, Eamon O’ Shea, Siobhan Fox, Kate Irving

**Affiliations:** 1 School of Nursing and Human Sciences, Dublin City University, Glasnevin, Dublin 9, Ireland; 2 Centre for Gerontology & Rehabilitation, University College Cork, Cork, Ireland; 3 Irish Centre for Social Gerontology, National University of Ireland, Galway, Ireland

**Keywords:** Dementia, Respite, Service development, Person-centred care, Meta-ethnography

## Abstract

**Background:**

Respite services provide a break in the caregiving relationship for people with dementia and their carers, however they are often under-used and service acceptability can be low. This study aims to understand key stakeholders’ experiences of respite services for people with dementia, with a view to informing respite service development.

**Methods:**

A systematic search was conducted of the Pubmed/MedLine, Embase, Cinahl, PsychInfo, Scopus, Web of Science, and Cochrane databases (1980–2016, English) with fixed search terms relating to ‘respite’ and ‘dementia’, following PRISMA guidelines. Noblit and Hare’s approach to meta-ethnography was employed. Key concepts were identified across the papers and reciprocal and refutational translation techniques were applied to primary studies; findings were synthesized into third order interpretations and finally, a ‘line-of-argument’ was developed.

**Results:**

In total 23 papers were reviewed, which described 20 independent samples across 12 countries. The views of 889 participants were synthesized (13 people with dementia, 690 carers, 44 ‘service providers’, 52 frontline staff, 70 managers, 12 volunteers, six academic/policy-makers, and two independent consultants). Five key concepts were identified and outlined i.e. 1) the transition to service use 2) expanding organizational capacity 3) dementia care quality 4) building a collaborative care partnership and 5) dyad restoration. There was broad agreement around the key areas for service development across the range of stakeholders (flexible and responsive person-centred care, meaningful activity for people with dementia, enhanced client-service communication and informational support). However, there was clear divergence in stakeholder perspectives around the barriers to implementation of such developments. Organizational tension was evident between frontline staff and management in respite services, hindering the cultural change necessary to facilitate service development in line with dyad’s needs and preferences.

**Conclusion:**

Respite services must surmount internal organizational barriers to change, and cultivate a collaborative solution-focused care culture, which acknowledges the centrality of the dyad and their care preferences. Future research should explore the development of alternative/modified community respite service models, which have greater capacity to be responsive to the needs of each individual dyad. The perspectives of people with dementia must be included in research in this area going forward.

**Trial registration:**

PROSPERO Registration Number: CRD42016050191.

**Electronic supplementary material:**

The online version of this article (10.1186/s12877-017-0676-0) contains supplementary material, which is available to authorized users.

## Background

Dementia is typically defined clinically as a syndrome of cognitive decline that is sufficiently severe to interfere with social or occupational functioning [1]. In 2010 it was estimated that there were 35.6 million people living with dementia globally, with numbers projected to rise to 65.7 million by 2030, and to 115.4 million in 2050 [2]. The need for appropriate and acceptable health and social care services for people with dementia is increasing as the population ages and more people are diagnosed with dementia. The experience of this condition can be challenging for people with dementia and their carers, and can make the dementia caregiving relationship difficult to maintain in the longer term. Given that people with dementia have a stated preference to ‘age-in-place’ [3], and that institutionalisation is costly and associated with a range of adverse outcomes [4–6], it is important to ensure that services are in place that can support people with dementia and their carers to achieve ageing-in-place, if that is their preference.

‘Respite’ services are often cited as an essential support, which can delay or prevent institutionalization, and are commonly defined as providing a break in caregiving for the carer [7]. Respite models can include residential care, day care and in-home care, and services can differ considerably across a range of parameters including: provider; setting; duration; and the quality and components of care. Carers’ service needs may differ on a number of broad life circumstances [8], including age, cohabiting, carer health status, other dependents, the nature of their relationship to the person with dementia and their employment status. However, there is evidence that along with access and availability issues, and psychosocial barriers to attendance, there can be a significant lack of client trust in existing respite services and the quality of dementia care provided [9, 10]. These findings indicate that respite services may not always provide care that is acceptable to its clients (i.e. people with dementia and their carers).

To date there has not been a systematic review of the literature which can inform respite service development in relation to dementia. Therefore, in order to understand how we can feasibly develop respite services, the research question guiding this review was: What are key stakeholders’ (i) experiences of respite services, and (ii) perspectives on respite service development, in relation to dementia? It is important to consider the perspectives of the range of key stakeholders (clients [i.e. people with dementia, carers], respite service staff and management, other healthcare professionals with a stake in respite services and policy makers) and to marry these together to understand not only how respite services should be developed in line with client preferences, but also the factors that might influence the implementation of such developments in complex health and social care systems.

## Methods

The review followed the Centre for Reviews and Dissemination guidelines for conducting systematic reviews in healthcare and is reported in line with the ‘enhancing transparency in reporting the synthesis of qualitative research’ (ENTREQ) statement (see Additional file 1: Table S4 for completed checklist) [11].

In line with Noblit and Hare’s [12] approach to meta-ethnography, the first step was to clearly state the specific research question. The second step was to determine the inclusion/exclusion criteria (see Table 1) and devise a search strategy to identify studies which can speak to the research question.

**Table 1:** Inclusion and exclusion criteria guiding study selection

Inclusion criteria
∙ Primary qualitative studies focused on respite services as they relate to dementia. ∙ Mixed methods research with a distinct and clearly reported qualitative element. ∙ Only studies with descriptions of the i) data collection and ii) data analysis procedures will be included. ∙ Studies employing surveys will be included if they collect qualitative data relating to the research question and meet other inclusion criteria. ∙ Participants with dementia and their carers must be community-dwelling. ∙ Published in English in peer-reviewed journals.
Exclusion criteria
∙ Quantitative studies with no qualitative element. ∙ Qualitative studies which do not include the perspectives of key stakeholders e.g. studies employing observational techniques only. ∙ Studies which include or are focused on older adults generally (not just people with dementia) and/or their carers. ∙ Studies which are not peer-reviewed e.g. reports, theses. ∙ Studies not reported in English.

### Search strategy

A search was conducted of the Pubmed/MedLine, Embase, Cinahl, PsychInfo, Scopus, Web of Science, and the Cochrane databases (date parameters 1980–2016). The search strategy aimed to identify all peer-reviewed literature relating to the research question. Google and google scholar were also searched to locate any unindexed peer-reviewed literature relevant to the research question. A hand search of the reference lists of the included studies, and of other relevant reviews, was also conducted as a ‘back search’, while the ‘cited by’ function of google scholar was used to ‘forward search’ for articles that have cited the included studies, and have relevance to the present research question.

### Search terms

The following terms were used in the search strings: Respite care (MeSH)*, respite*, day care (MeSH), day-care (MeSH)*, residential respite*, in-home respite*, Dementia (MeSH)*, Alzheimer disease (MeSH)*, alzheimers*, cognitive impairment*.

In Boolean operators:

Dementia **OR** Alzheimer disease **OR** Alzheimer’s **OR** cognitive impairment **OR** older adults **OR** frail elderly.

**AND**

Respite care **OR** respite **OR** daycare **OR** day care **OR** residential respite **OR** in-home respite **OR** in home respite.

### Data collection and quality appraisal

**Fig. 1:**
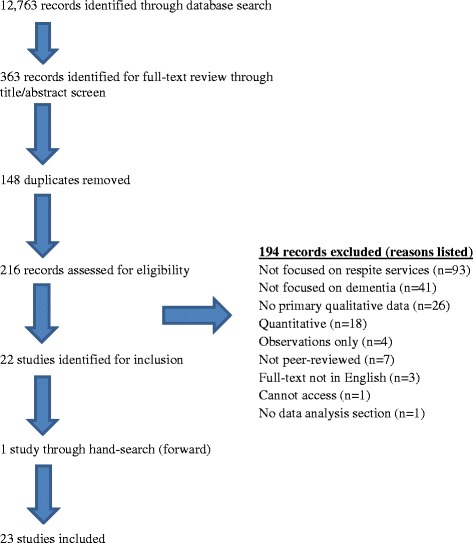
PRISMA Flow Chart of Study Selection Process

Two authors independently screened abstracts and full-texts, and subsequently reviewed eligible full-text articles for inclusion based on the above inclusion/exclusion criteria. Where articles were rejected, reasons for rejection were recorded and are outlined in the PRISMA flow chart (see Fig. 1). Data items extracted include information about the publication (date, authors, country, study aim(s)), study eligibility (design, methods, and analysis), respite model, participant characteristics, and raw data (in the form of themes and/or quotations). Data was managed using NVivo 11.

The quality of the studies was assessed using the Evaluation Tool for Qualitative Studies (ETQS) [13]. This tool was employed with a view to assessing the validity, robustness and transparency of each study. According to Hannes et al. [14], the ETQS is preferable to the widely-used CASP tool [15], as it provides more detailed instructions on how to interpret the evaluation criteria. It contains the following overarching sections, each with a template of key questions to guide the appraisal: phenomenon studied and context issues; ethics; data collection, analysis and researcher bias; policy and practice implications [13]. The quality assessment was conducted independently by two authors (EOS, SF). The quality assessment was not used to exclude studies in this review, given that the findings reflect the quality of the written report, rather than the actual research process in many cases [16, 17].

### Data analysis and synthesis

The analysis and synthesis were guided by Noblit and Hare’s meta-ethnography approach [12], and informed by recent methodological papers on this ‘evolving’ approach by France et al. [17] and Toye et al. [18]. Meta-ethnography is a form of interpretative synthesis that can be used in the reviewing and evaluation of qualitative research studies [12]. This method was chosen as it moves past the simple summarising of primary data. It is used as an inductive method which serves to compare, translate and integrate concepts across studies, while also attempting to preserve the context and interpretive properties of the primary data [16, 19]. This method is often used to create new concepts and theories, or develop upon existing ones [18]. It is increasingly being employed in health research, in particular in relation to patients’ experiences of illness and care [16].

Initially, papers were read and re-read to identify information on the study context. Subsequently, the ‘results’ and ‘discussion’ sections of the included studies were coded inductively for meaning, as they related to the research question; codes were attached to meaningful segments, as opposed to strict line-by-line coding. A second author (SF) independently coded over 20% of papers (5/23) for quality assurance purposes and differences were settled through discussion.

These codes and the corresponding raw data were then compared, using (i) reciprocal translation (recognising reoccurring themes/concepts across studies) and (ii) refutational translation (recognising themes/concepts that are dissimilar across studies, but cannot be explained by contextual factors). A constant comparison approach was employed to achieve this; included studies were considered in chronological order by date of publication, and the codes and concepts in each subsequent study were compared against those in all the studies that preceded it. This continued through an iterative process until no new translations could be made.

**Fig. 2:**
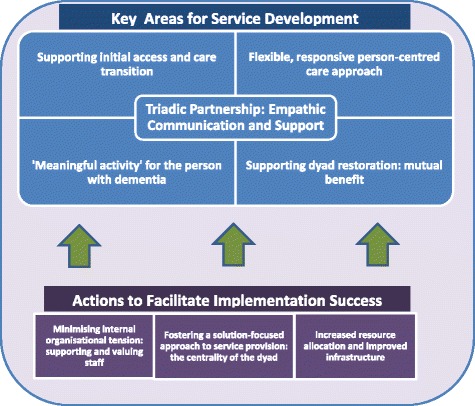
Conceptual model of the synthesized stakeholders’ perspectives on respite service development and actions that might facilitate implementation success

In terms of the data synthesis, the focus of our ‘third order construct’-building (i.e. present reviewers’ interpretations), was based largely on marrying the ‘second order’ constructs (the original authors interpretations of the primary data), with our interpretations of first order constructs (participant raw data), all while bearing the original study context in mind. The synthesis of the present findings lead to 23 final translations (see Additional files), which informed the final line of argument and the conceptual model (see Fig. 2). NVivo11 was used to organise and manage the data, codes and concepts during the initial translation stage. The final translations (and the corresponding data and concepts) were extracted and synthesized using a matrix in Microsoft Excel. The synthesis was conducted by EoS and KI, in consultation with the other co-authors.

The seven sequential steps that were followed in relation to the data synthesis element of this meta-ethnography are summarised in Table 2.

**Table 2:** Key steps involved in conducting data synthesis following data extraction

Step	Process
1	Active reading of the studies to understand the context, to appraise study quality and to extract relevant data
2	Themes/ concepts were identified in the ‘results’ and ‘discussion’ sections of papers from both i) participant raw data (first order constructs) and ii) the authors’ interpretations of this (second order constructs) and these were coded
3	Second order constructs and the assigned codes were compared and contrasted within and across studies in NVivo11
4	Overall key concepts were identified (with narrative summary) and extracted to excel to be outlined in a matrix
5	The relationship between i) each study and ii) each key concept was examined, with the original context of each in mind
6	Studies were ‘translated’ (reciprocally and refutationally) into one another and the similarities/differences identified enabled the development of a conceptual model/theory/etc. which accounted for these based on the original study context. Here the original language used by authors is retained (fidelity to original meaning/context)
7	A synthesis of translations allowed for the building of third order constructs and the ultimate development of a line-of-argument synthesis. Active re-reading of the original studies was conducted to verify the appropriateness of the synthesis, prior to write-up

## Results

A total of 12,763 records were identified through the database search (see Fig. 1 for flow-chart). After the title/abstract screening process, and removal of duplicates (*N* = 148), 216 full texts were reviewed. We excluded 194 records at this stage. One further paper was identified through a forward hand-search of the 22 included papers. Thus, we included 23 papers in total.

### Characteristics of included studies

In total 23 papers were reviewed, describing 20 independent samples (17 entirely qualitative studies, three mixed methods studies). The views of 423 participants were synthesized from the 17 independent qualitative studies, including 13 people with dementia, 224 carers, 44 ‘care providers’, 34 ‘frontline staff’, 53 ‘managers’, 12 ‘volunteers’, six ‘academic/policy-makers’, and two ‘independent consultants’. Of the three unique mixed methods (four papers) studies reviewed, the perspectives of a further 466 carers (open-ended survey responses), 18 ‘frontline staff ‘(semi-structured interviews) and 17 ‘managers’ (semi-structured interviews) were synthesized. The studies were conducted across 12 countries, spanning four continents (see Additional file 2: Table S5 for study characteristics).

### Quality assessment

The summary of the quality assessment conducted using the ETQS is illustrated in Table 3. Overall, 10 studies were considered medium-high, 10 studies medium, and 3 low-medium quality in relation to methodological reporting transparency. No articles were excluded on the basis of the quality assessment.

**Table 3:** Summary quality assessment of included studies

First author, year	Phenomena under study	Theoretical framework/orientation	Setting	Sampling/Recruitment	Depth/Breadth of Perspective	Ethics	Data collection	Data analysis	Positionality/Reflexivity	Policy/Practice Implications	Quality assessment
Brataas, 2010 [31]	√	√	√	√	√	√	√	√	X	√	Medium-High
Cahill, 2003 [32]	√	√	√	X	√	X	X	X	X	√	Low-Medium
de Jong, 2009 [30]	√	√	√	√	√	√	√	√	X	√	Medium-High
Donath, 2009 [41]	√	√	√	X	X	√	√	X	X	√	Medium
Donath, 2011 [40]	√	√	√	X	X	√	√	X	X	√	Medium
Gilmour, 2002 [36]	√	√	√	X	√	√	√	X	X	√	Medium
Gústafsdóttir, 2014 [22]	√	√	√	√	√	√	X	X	X	X	Medium
Hochgraeber, 2015 [27]	√	√	√	√	√	√	X	√	X	√	Medium-High
Holm, 2003 [29]	√	X	√	X	√	X	X	X	X	√	Low-Medium
Huang, 2016 [28]	√	√	√	√	√	√	√	√	X	√	Medium-High
Jansen, 2009 [26]	√	√	√	√	√	√	√	√	X	√	Medium-High
Kirkley, 2011 [38]	√	√	√	√	√	√	√	√	X	√	Medium-High
McGrath, 2000 [34]	√	√	√	√	X	X	√	√	X	√	Medium
O’Connell, 2012 [20]	√	√	√	√	X	√	√	X	X	√	Medium
Parahoo, 2002 [33]	√	√	√	√	√	X	X	X	X	√	Medium
Perry, 2001 [42]	√	√	√	√	X	X	√	X	X	√	Medium
Phillipson, 2011(IH) [23]	√	√	√	√	√	√	√	√	X	√	Medium-High
Phillipson, 2011(RR) [24]	√	√	√	√	√	√	√	√	X	√	Medium-High
Phillipson, 2012(DC) [25]	√	√	√	√	√	√	√	√	X	√	Medium-High
Robinson, 2012 [21]	√	√	√	√	√	√	√	√	X	√	Medium-High
Strang, 2000 [37]	√	√	√	X	√	X	√	X	X	√	Low-Medium
Upton, 2005 [35]	√	√	√	X	√	√	X	X	X	√	Medium
Woolrych, 2013 [39]	√	√	√	√	√	X	√	X	X	√	Medium

X = Not Clear/Not Methodologically Sound; √ = Clear/Methodologically Sound

Quality assessment categories: Low-Medium = 6 ≥ X, Medium 3-5X, Medium-High 2 ≤ X

## Translation

This analysis has highlighted five key concepts, comprised of 12 subthemes that outline the experiences of key stakeholders in dementia in relation to respite services, and their perspectives on respite service development.

The overarching concepts are presented in the following order (not indicative of concept salience):

1. Transitioning to service use
2. Expanding Organizational Capacity
3. Dementia Care Quality
4. Building a Collaborative Care Partnership
5. Dyad Restoration

Both first order (raw primary data i.e. direct participant quotations) and second order interpretations (primary study authors’ interpretations of the primary data) are employed to support the analysis below. First order interpretations are indicated in italicised quotations, while second order interpretations are indicated by non-italicised quotations.

The final synthesis of the concepts from the range of stakeholder viewpoints presented below, led to the development of a model (Fig. 2) which illustrates 1) the crucial areas identified for improvement across respite service models, as they relate to people with dementia, and 2) the perceived barriers and facilitators to implementing these developments, primarily from the perspectives of clients (almost entirely carers’ views), and service staff and management.

### Transitioning to service use

#### Timely access to services

Accessing respite services was considered challenging for carers across a number of studies, both from the perspectives of carers [20–25] and service providers [26, 27]. The availability of appropriate services was generally deemed poor by carers [23]: “*There are different levels of care but nothing is quick ... and there are not many places when you are actually faced with trying to find one to use*” [23]. Carers noted that availability was often poor even in what they considered “emergency situations” [23, 24]; this indicates a possible disparity between client and service views of what constitutes an ‘emergency’, influenced by the poor availability of respite services: *“...if I rang today and said I needed someone today because I’m going to the hospital…they’d say no we don’t have anyone”* [24].

Carers experienced significant difficulties navigating the health and social care system, particularly in terms of accessing respite care, including identifying personnel who could connect them to available respite services and help them to identify their support needs [21]*.* Providers agree that the process of gaining access should be more transparent, and suggest that minimising bureaucracy and assigning a “central point of contact” to assist families would be beneficial [26, 27]. Providers therefore see the service access problem to be rooted primarily in the under-resourcing and the infrastructural inadequacies of the system. They note, for example, that carers frequently present at hospitals to gain access to services as they reach crisis point, having failed to traverse the fragmented system [26]. However, some providers admitted some personal culpability at the service level, indicating that even they, staff working within the system, have a “lack of knowledge about the available services” [27].

While cost sharing did not feature as a major concern for carers, some carers do reference cost as a barrier to use [20, 28]. Service providers agree that cost sharing sometimes acts as a barrier to access, particularly for residential respite services [26]. However, other providers believe that reducing user costs would undermine the value of services for stakeholders and might ultimately adversely affect public perception of care quality [27].

A particularly common access concern for carers was the provision of safe and reliable transportation to and from community-based day services [20, 25, 27, 29–31]: “the fact that they were able to provide transport to the centre and home again…without that it all would have been too hard” [25]. Despite the centrality of transport for carers, some service providers deemed this to be extremely resource-heavy and therefore not always feasible [26, 32]: “You have to get them there in a reasonable time; however, the costs to take them there may prevent that” [26].

#### Service acceptability and negotiating service refusal

Carers describe a ‘settling in’ process whereby services are gradually introduced in an effort to make the person with dementia feel safe and secure in the midst of a significant care transition [21, 33]. During this “trial and error” period, carers focus on appraising the service-client fit [21], in terms of disruption to routine [20, 22, 33], personality factors (e.g. introverted vs extraverted, [28]), sex (e.g. day services as unacceptable to some men) [25], early versus later onset dementia (e.g. day services as unacceptable for early onset [25, 33], dementia ‘stage’ [28], staff ability to manage complex behavioural and physical needs [23, 25, 34] and the service model [33, 34], with a view to assessing the initial acceptability of the service.

Carers report that service refusal on the part of the person with dementia can be a significant relational obstacle in the transition to service use [21, 23, 24, 28] and considerably adds to carer strain, sometimes making service use “more trouble than its worth” [24]. Some carers have suggested that this refusal is impacted upon by factors including stigma and fear of social evaluation [21, 28], (e.g. others *“thinking he’s a dill”* [21]). Carers’ responses to service refusal were diverse, spanning from acceptance and acquiescence (particularly carers who felt high guilt [21]), to frustration and despair [21, 25, 28, 35]. Other carers endured the initial conflict and persisted with service use regardless [21, 25]. Carer’s suggestions for negotiating service refusal were around reassurance and “promoting feelings of safety and security” and “not giving up” [21], as well as explaining the potential benefits of service use to the person with dementia [34]. Regarding the perspectives of people with dementia who use day services, initial ambivalence and reluctance in relation to service use diminished when they were made to feel secure about their “cognitive losses”, and came to trust that the staff were predictable and reliable [31]. Providers’ perspectives on the potential role of services in mediating service refusal are not clear. However, providers do acknowledge the potential role stigma has in dementia respite service non-use, believing that the stigma is in relation to service use itself, and that the solution to this lies in improving the public image of respite care [27].

#### Carers’ negative beliefs about service use

The transition to service use can also sometimes be complicated by carers’ negative beliefs about service use, and their concerns about the safety of, and the outcomes for, the person with dementia, both from the perspectives of users and non-users [23, 25, 35, 36]. Carers worried that service use would ultimately cause deterioration in the person with dementia, thus intensifying care strain [23, 35, 36]: *“… he might be a lot worse mightn't he? ... So l mean there's not much point in that is there if I'm going to suffer afterwards…”* [35]. Additionally, service use was synonymous with failure to cope [25], and/or to fulfil their duty to care [24], leading to feelings of guilt for the carer [23, 28, 35], particularly for spousal carers. Carers also felt that it signalled that the need for permanent placement in long-term care was imminent [23, 33]. However, carer’s sometimes had no choice but to overcome such beliefs about service use, as their need for support became insurmountable [25]; “*I couldn’t allow that thought to stay with me because I knew I had to survive, I had to have help.”*

Furthermore, carers sometimes either don’t perceive, or accept, their own need for a break necessitating service use [23, 28, 33]. However, as the dementia condition progresses and their coping resources deplete, they are forced to recognise this need [23, 25, 33, 37]. In particular, children of people with dementia may be forced to acknowledge this need sooner than spousal carers, because of a range of other responsibilities consistent with their life stage, including being in employment and raising their own children [33]. Social, familial and professional ‘referents’ can serve to reinforce and validate the legitimacy of the carer’s need for a break [23, 25, 26, 35, 37]:*“…they* [day centre staff] *helped me realise that it was normal to need a break”* [25]. Conversely, other family members can potentially complicate the primary carer’s beliefs about the need for service use [23, 28, 33], particularly if their cultural values favour family-managed care [28, 33]. Notably, service providers did not reference this aspect of the transition to service use.

### Expanding organizational capacity

#### Resources & infrastructure

According to care providers, one of the key barriers to respite service development and improved care quality is human and fiscal under-resourcing [26, 32, 38]: “*If we had more money, more time, and more staff, that’s the only way I think we could make it more person centred*” [38]. Service providers feel that a lack of resources is preventing them from providing good quality care [20, 26, 36]. In terms of infrastructure, service providers state that the lack of appropriate and acceptable community and in-home services for people with dementia impedes the achievement of respite for some carers: *“I don’t always think that the respite options match the respite needs”* [26].

Both carers and providers have indicated that the design of the built environment of day services and residential respite services should be non-institutional, safe and serve to enhance the wellbeing of the person with dementia [20, 25, 28, 29, 32, 39]. Providers note that design features including inadequate space, orientation cues, and lighting and heating can increase behavioural and psychological symptoms in people with dementia [32], while unsuitable facilities (e.g. confined shower space, poor water supply, no handrails, [32]) can impact patient safety and staff strain [32, 39]. Some providers had ideas for improving the built environment, but believed that such improvements hinged on receiving increased funding [32].

#### Clinical governance

Both service providers and carers highlight governance issues in respite service planning and delivery [20, 25, 30, 32, 39]. For example, some models of respite (e.g. in-home) may require more regulation, safeguarding and monitoring to ensure high quality care provision and staff performance [29, 30]. Service providers note that the lack of training requirements for staff, particularly for in-home care, impacts care quality and continued service use: *“We rarely get people who understand dementia and have personal care skills*. *.*. *and sometimes when people do come in they’re not trained, and they don’t do a good job, and they make it worse so the clients don’t use home care”* [26]. Along with improved training, measures should be implemented that increase staff accountability and responsiveness at the service level [30]. Some authors also argue that additional international and national guidance is needed to inform the delivery of ‘best practice’ dementia respite for healthcare professionals [20, 25, 32].

Continuity of care is an essential issue in relation to respite services [24, 26, 27, 29, 30, 33, 36, 39], and can influence access, length of stay and the capacity of the service to deliver individualised care [39]. Management hold that “service co-ordination necessitates ongoing cooperation and communication between formal carers across all domains of care” to foster effective cross-organizational working. However, frontline staff indicate that, from their perspective, “bed-blocking” is one of the key barriers to continuity, and that this must be rectified at the systems-level (i.e. fragmentation and bureaucracy [“ticking boxes”]) [39]. Poor care co-ordination can also affect the client’s perspective of the utility of the service; carers want evidence of collaboration between all professionals involved in the care of the person with dementia [20, 29, 30, 33].

#### Facilitating organizational change

Fostering organizational change in respite services is a challenging task for service managers, particularly in relation to implementing person-centred care [38, 39]. Part of the role of management is to foster the “natural potential of staff” in terms of implementing this approach [38]. Staff must feel supported psychosocially and professionally [27, 38, 39], and in terms of their dementia education/training needs [26, 38], in order to provide person-centred care. However, managers are often feel powerless to lead on cultural change [38, 39], *“…you just have to pray they* [staff resistant to change] *take early retirement”* [38]. Interestingly, managers themselves were “not always aware of their own training needs”, making it difficult for them to lead on such change [38].

Flexibility and responsiveness in care provision is one of the main developments that both frontline staff and carers deem important across respite service models [20, 24, 27, 28, 30, 32, 39]. However, staff discuss the barriers to this in relation to organizational bureaucracy (“*hitting brick walls everywhere you turn*”), and how the absence of decision-making autonomy, and “risk-averse” managerial culture sometimes leaves staff feeling demoralised, powerless and incapable of implementing service improvement initiatives [38, 39]. Conversely, carers discuss this primarily in relation to dementia care quality and the staff approach to dementia care, which will not be outlined below.

### Dementia care quality

#### The care approach

Carers were clear that they expected staff across respite service models to deliver individualised care that demonstrates affection, friendliness, empathy and respect towards people with dementia [20, 21, 24, 28, 33, 36, 40], while also maintaining their ‘professional dimension’ [24, 36]. For some carers, good quality care was about treating the person with dementia “*like a human being*” [24], while for others it was more than this; it was about taking a more personalised approach that considers the “uniqueness” and “individual needs” of each person [21, 28, 33]. An example of a satisfactory approach to care was concisely summarised by one daughter: “*…they sent someone who was wonderful with mum ... she totally got it. She attended to mum’s physical needs ... but also in talking with her about her life ... engaging with her”* [24]. People with dementia also expressed an appreciation for “caring” staff [29] that “displayed attitudes of goodness, understanding and respect” [31].

Person-centred care was also important for service providers with some evidence of staff discussing people with dementia and their care in a manner consistent with personhood concepts and ideals [27, 29, 32, 38]. However, the practice of person-centred care can be challenging and, more often than not, services superficially label their care ‘person-centred’ without actually delivering this in practice [38], indicating that there is ambiguity around the meaning of what person-centred care is for some providers. For frontline staff, this more personalised approach to care requires dementia-specific training, while others believe that it would be facilitated by improved integration of services (and subsequent care continuity) at the organisational level [39].

#### Care components

While both carers and care providers noted that assistance with personal care, activities of daily living and medication monitoring were important care components [24, 26, 34], the most valued care component in terms of service development from the carer and provider perspective, was engaging the person with dementia in ‘meaningful’ activity [20, 24, 27, 29, 30, 32, 33, 37, 40–42]. Carers perspectives differed around what constitutes ‘meaningful’ activity for people with dementia [30]. Some carers valued physical activities e.g. walking [24, 27, 30, 37, 40, 41] and/or therapeutic and rehabilitative activities (e.g. directed at speech, cognition, reading, the arts) [24, 27, 30, 37, 42] [stimulation]), while carers in one study believed activities should not be imposed on the person with dementia [30]: *“Of course you have a day program and everything but, but if the man doesn’t want to do anything, then let him”.* For other carers, ‘meaningful’ activity was whatever the person with dementia deemed enjoyable [20, 29]. Therefore, while some carers prefer a prescribed activity programme that may maximise health outcomes and enhance functional abilities, other carers valued a tailored person-centric approach [20, 29, 30, 33] that engages the person with dementia and makes them feel “worthwhile” [25]. For people with dementia, activities that gave them a sense of “belonging” were considered important and “promote a good mood” [31]. However, according to service providers, because physical care (e.g. “continence care” and “bathing”) is hugely time-intensive, especially as the dementia condition progresses, staff are often limited in the time available to deliver non-physical care components [26].

### Building a collaborative care partnership

#### Empathic client-service communication

For carers, the development of trusting care relationships between the dyad and the service was underpinned by empathic communication about the person with dementia and their care [20–22, 24, 30, 36]. Perceived poor communication with services makes it difficult for carers to relinquish the carer role and achieve a positive respite experience [36] and carers believe this leads to adverse outcomes [20, 36]. Amongst carers who were satisfied with care quality, it was evident that they felt that their views had been solicited and valued by staff [21, 22, 30, 36]. Trust in care quality was amplified when carers felt that staff were asking “*the right questions*” about the person with dementia and their care [36], and when staff were seen to be developing a positive relationship with the person with dementia [24]. A designated point of contact was considered a substantial benefit in terms of dyad-service communication by carers and frontline staff [21, 27, 30]. There is minimal research from the perspective of the person with dementia, but people with dementia valued being included in care decisions and reported that they valued listening, and being listened to, by staff [31].

From the staff perspective, communication is also considered essential for relationship-building [26, 39]. “*Once they get to know you, they start to trust you. Communication is the big thing*” [39]. However, the development of a trusting client-service care relationship requires a dedicated time commitment which staff feel must be better supported at the organizational level [39], again highlighting the resource issue. Furthermore, service providers noted that client-service communication regarding care should be collaborative, not directive, and staff should seek to understand the carer’s perspective on meeting the care needs of the person with dementia [26, 36].

#### Meeting carer’s informational support needs

Carers also value informational support and advice from staff within services in terms of developing their understanding of dementia, improving the quality of care that they themselves can provide at home, and their own capacity to cope in the carer role [24, 30, 33]. Carers want information about managing behavioural and psychological symptoms, as well as on safety issues e.g. “falling, handling drugs, the danger of gas stoves, and arranging aids and adaptations to the home” [30]. One carer noted that the in-home service he received would have been more supportive, if he had been given much-wanted advice and education in relation to dementia care [24]: *“I would really like, not just a well-trained caregiver, but an external adviser. I am the only person that looks after my wife most of the time, and it would be invaluable to be able to say to that person, if they had the knowledge, well you know, how do you think she is going, what do you think her needs are ... do you think we are meeting her needs”* [24].

### Dyad restoration

#### Mutual benefit

Some carers conceptualise respite, not just as a service, but as an experience and/or an outcome i.e. a restorative psychological and physical break from caregiving, which they can achieve when they perceive that both sides of the dyad benefit mutually from service use [34, 37, 42]. For carers, a number of service and client (psychosocial, occupational) factors impact upon the benefit they experience from service use. In terms of service factors, the length/duration of the respite period and the perceived quality of care are important [20, 24, 33, 37]. There is divergence in findings around carer preferences for length of time in respite care [34, 35, 37]; some carers prefer frequent short intervals (e.g. day service, in-home models) which facilitate them in keeping on top of chores, while others prefer longer intervals (i.e. residential/ overnight models) as they feel this extended block of time better allows for revitalisation [37]. However, there is general convergence that carers in both day service and in-home models would prefer longer breaks [20, 24, 33, 37]. Regarding care quality, it was important that the carer trusted that the person with dementia was being appropriately cared for [22, 24, 36]. Perceiving that the person with dementia was safe and satisfied, allowed carers to relinquish the caregiving role temporarily [36] and alleviated carer guilt around ‘abandoning’ the person with dementia [20, 24, 34]. Carer restoration was, in part, determined by how carers chose to occupy their time during service use [25, 33–35, 37, 42]. Ideally, this should be with absorbing activities (e.g. socialising, hobbies) and not just urgent or menial errands [34, 35, 37, 42]. A number of study authors concluded the need for services to deliver individualised interventions to informal carers, to help them to overcome the psychosocial and relational barriers to achieving a psychological respite experience [21, 24, 34, 35].

#### The post-respite evaluation

After service use, carers continually evaluate the benefits of service use for the person with dementia, to determine the utility of the service for dyad restoration. This is assessed through observing the stated satisfaction/dissatisfaction of the person with dementia, across respite models [20, 22, 29, 36, 37, 42] and monitoring the post-respite outcomes for the person with dementia [20, 23, 35–37, 42]. Where poor outcomes were evident, carer’s were reluctant to use the service again, particularly in relation to residential respite: *“…after two weeks she stopped walking, lost hair from being left in bed all day. … was very upsetting to my wife and she certainly slipped back in her health. I don’t know if I could use this again*” [20]. Those carers who were satisfied that service use was mutually beneficial perceived psychosocial, functional and cognitive gains for the person with dementia [20, 21, 25, 30, 34, 42]. Finally, for people with dementia, a beneficial day care experience was described as something that fostered vitality in them, and increased their self-worth, happiness and energy levels [31].

## Synthesis

Each of the five concepts in this study was linked with a number of third order interpretations: Transitioning to Service Use (*n* = 6), Expanding Organizational Capacity (*n* = 6), Dementia Care Quality (*n* = 3), Building a Collaborative Care Partnership (*n* = 5) and Dyad Restoration (*n* = 3) (see Additional file 3: Table S6). The third order interpretations were synthesized to develop a line of argument (see Fig. 2 for conceptual model) in relation to key stakeholders’ experiences of respite services and their perspectives on service development. This line of argument will now be outlined.

There is broad agreement across the range of stakeholders regarding the key areas for respite service development. There is consensus around improving access and better supporting the transition to service use, as well as providing more flexible and responsive person-centred care and tailored meaningful activity. There was also strong support for ensuring that both sides of the dyad benefit mutually from service use. For carers, their ability to achieve a restorative respite experience is largely dependent on the perception that the service recognises the centrality of the person with dementia and their care needs, and that staff within the service are willing to partner and collaborate with the dyad to understand these needs. Carers indicate that effective client-service care collaboration is underpinned by empathic communication and validation. Building this type of supportive care partnership serves to reassure the carer about the service-client fit in terms of the care approach and components. Ultimately, it is this that facilitates carers in achieving a restorative physical and psychological break from caregiving through service use.

However, the findings also indicate that there are divergences among client, staff and management perspectives on the key barriers and facilitators to implementing such respite service developments. Carers predominantly locate their preferences regarding service development at the service-level, in terms of what frontline staff can do to improve respite service provision (care approach, activities, communication, informational support etc.). However, there is some discordance between staff and management perspectives on implementation barriers. Staff tend to believe that service development is largely dependent on building organizational capacity at the systems-level (e.g. increased resources and improved infrastructure). Staff also point out that bureaucracy and a risk-averse managerial culture does not support them to implement developments at the service-level. While management do acknowledge the need for expanded capacity at the systems-level, they also perceive staff reluctance to engage with cultural change as a substantial barrier to service development at the service-level. This within-service conflict can serve to immobilise development initiatives. It is important that services focus on minimising bureaucratic and risk-averse organizational values and structures, and foster a culture that is collaborative, values staff, and supports them educationally and psychosocially to enact change in line with client preferences.

## Discussion

This is the first study, to our knowledge, to systematically review and synthesize the qualitative literature on key stakeholders’ experiences of respite services, as they relate to people with dementia, with a view to informing service development. The findings underline a number of areas for improvement, on which there was broad agreement across the range of stakeholders: improved access and transition; flexible and responsive person-centred dementia care; ‘meaningful activity’ for the person with dementia; empathic client-service communication; and restorative care for both sides of the dyad. However, the findings indicate that implementing such developments in respite services is an extremely complex undertaking and one that may require a multi-faceted implementation approach, underpinned by wider organizational cultural changes within and across services, as well as enhanced resource allocation.

### Developing respite services: Implementing person-centred dementia care

The majority of studies reviewed here recognised the importance of person-centred dementia care in respite services. However, many barriers to its delivery were clear, across the range of stakeholders. It is clear that organizational cultural change is perhaps the most important consideration here in terms of understanding how we can implement such developments in respite services, in line with client needs and preferences.

Dupuis et al. [43], describe a culture change initiative, Partnerships in Dementia Care (PiDC), which is being rolled out in long-term care settings in Canada, and which is informed by the integrated theoretical and philosophical underpinnings of a number of approaches to culture change. It is possible that PiDC could be a useful framework to conceptualise how more bottom-up implementation approaches could be successful and sustainable in respite services that currently are at a stalemate in terms of shifting the care culture towards a more responsive and person-centred approach, in line with client preferences. The fact that the PiDC initiative advocates for a ‘bottom up’ implementation approach is important in relation to the findings of this synthesis, which indicates that the top-down approaches often employed in respite services (in which organizational values and attitudes in relation to dementia care are imposed upon frontline staff, often in the absence of the necessary supports and education) do not work well, and may be at least partially accountable for the organizational tension evidenced in this review.

Bottom-up approaches such as PiDC advocate for a more relational basis to culture change, and this seems important on at least two levels based on the present findings, i.e. within-service relations, and client-service relations. The principles of this framework highlight the importance of adopting a relationship-based approach through collective decision-making, valuing abilities, respecting others, accountability and shared responsibility, and focusing on ‘the process’ (which is primarily about empathic communication within and between all stakeholders, ongoing reflection and open dialogue). In terms of applying this framework, the authors note that the partnerships framework can be conceptualised as being about i) ‘working collaboratively’, ii) ‘thinking and doing differently’ and iii) ‘re-imagining new possibilities’, in relation to dementia care. Including the voice of the person with dementia and their carer in this process is absolutely crucial to imagining any new possibilities.

### ‘Respite’ service development – What’s in a name?

According to cultural change initiatives, such as the PiDC framework outlined above, ‘thinking and doing differently’, and ‘reimagining new possibilities’ are central modes of change. In relation to respite services, the term ‘respite’ which currently guides the planning, organization and delivery of services aiming to provide a break in caregiving for the carer, is not a useful term when considered in the context of person-centred dementia care [44]. This is because the baggage associated with ‘respite’ indicates that it is a term which only speaks to the experience and perspectives of the carer in terms of the dementia condition, the dyadic relationship and service use; in this way it is loaded with meaning that cannot speak to the experiences of the person with dementia, and therefore is fundamentally discordant with the principles of person-centred dementia care. We must consider re-imagining the terminology used to describe the aims of what are currently known as ‘respite’ services, to ensure that the nomenclature guiding service delivery is consistent with the principles of person-centred dementia care.

While any new nomenclature is up for debate, one possible re-imagination of the term ‘respite care’ is ‘restorative care’, which has more potential to highlight the importance of providing mutually-beneficial, personalised health and social care services that serve to enhance care relationships [44]. The weakness of the current literature in eliciting and articulating the views of the person with dementia is a cause for concern and needs to be addressed urgently.

### Strengths, limitations & future directions

Measures were put in place to maximise the quality of this review, including practicing reflexivity, employing two independent reviewers, and adhering to the PROSPERO protocol (registration number below). This study was conducted by an experienced multidisciplinary team (geriatric medicine, nursing, economics, social policy, psychology) with expertise in qualitative approaches. Consequently, we believe that the conceptual model (Fig. 2), synthesizing stakeholders’ perspectives on service development, provides the reader with a rich third-order interpretation of how improvements might be implemented in respite services for people with dementia. Another strength of this review was the number and diversity of stakeholders (family/informal carers, people with dementia, frontline staff, service managers etc.) and countries (12 countries, across four continents [Europe, Asia, Australia/Oceania, and North America]) included. The inclusion of multiple perspectives allows for a more holistic view of the topic of respite service development, including the factors that might influence implementation.

Nonetheless, the primary limitation, potentially affecting the validity of this review, if not the whole research community, is the absence of people with dementia in the primary studies; there were just 13 identifiable people with dementia represented here out of 889 stakeholders. Another limiting factor is that the findings of this review are based on studies published only in English, and studies conducted primarily in western countries. Therefore the findings may not represent countries with different cultures, models of respite provision, and/or low-middle income countries. The methodological quality of some of the studies included in this review was marred by a lack of transparency around the characteristics of the respite services, sampling, recruitment and data collection and analysis techniques. Future research in this area must comply with published reporting guidelines to ameliorate these issues.

Future research must also include the experiences and views of people with dementia in relation to respite services and service development. Other areas which require further research include the potential role of services in easing the transition to service use (e.g. mediating service refusal, validating carers’ respite needs) and facilitating a restorative experience for the dyad, understanding how best to minimise internal organisation tension and build a collaborative client-service care partnership, and exploring modified/alternative models of respite provision which are more responsiveness than traditional models.

## Conclusions

This review has found that key stakeholders are in broad agreement about a number of key developments which are necessary across service models, to improve respite care for people with dementia. These include improved access and transition; flexible and responsive person-centred dementia care; ‘meaningful activity’ for the person with dementia; empathic client-service communication; and restorative care for both sides of the dyad. However, divergent stakeholder perspectives around implementing such developments highlight that organizational cultural change is an extremely complex process, requiring a multi-faceted, relational, bottom-up approach for successful and sustainable implementation. Service managers must build organizational capacity together with frontline staff to facilitate meaningful restorative experiences for dementia dyads. This might achieved by taking steps to foster a collaborative solution-focused culture of care which acknowledges the centrality of the dyad and their care preferences. Future research should explore the development of alternative/modified ‘respite’ service models, which have greater capacity to be flexible and responsive to the needs of each individual dyad. Finally, the perspectives of people with dementia must be included in qualitative research in this area going forward.

## Additional files

Additional file 1: Table S4. The ENTREQ Reporting Guidelines Checklist. (DOCX 17 kb)

Additional file 2: Table S5. Characteristics of the Included Studies. (DOCX 21 kb)

Additional file 3: Table S6. Third-Order Interpretations Relating to Key Concepts. (DOCX 16 kb)

## Abbreviations

ETQS: Evaluation Tool for Qualitative Studies
PiDC: Partnerships in Dementia Care
