# Supplementary material for: Key stakeholders’ experiences of respite services for people with dementia and their perspectives on respite service development: a qualitative systematic review
Source: BMC Geriatr. 2017 Dec 7;17:282. doi: 10.1186/s12877-017-0676-0 (PMC5719558; doi:10.1186/s12877-017-0676-0)
Supplement: Supplementary file 2 — Characteristics of the Included Studies. (DOCX 21 kb) [file 12877_2017_676_MOESM2_ESM.docx]

**Table 5- Characteristics of the Included Studies**

| **First author, Year** | **Country** | **Study aim(s)** | **Method** | **Participants (N)** | **Respite Model** | **Data Collection (qual)** | **Data Analysis (qual)** |
| --- | --- | --- | --- | --- | --- | --- | --- |
| Brataas, 2010 | Norway | To understand how older adults with cognitive impairment perceive and experience daycare. | Qualitative | People with dementia; 2 males and 7 females (N=9). | Daycare | Semi-structured interviews | Narrative content analysis |
| Cahill, 2003 | Ireland | To understand service providers’ views on dementia-specific daycare. | Mixed Methods | 18 nurses, 17 branch chairpersons; (N= 35). | Daycare | Semi-structured interviews | Specific approach not stated |
| de Jong, 2009 | The Netherlands | To explore the needs and wishes of informal caregivers for the provision of skilled psychogeriatric day-care. | Qualitative | Family carers of PwD (4 spouses, 5 other); (N=9). | Specialist daycare | Semi-structured interviews | Thematic analysis |
| Donath, 2009 | Germany | To explore the views of caregivers of PwD about the quality of short-term residential care. | Mixed Methods | Carers of people with dementia; (N=254). | Residential respite | Open-ended survey questions | Content analysis |
| Donath, 2011 | Germany | To explore the views of family caregivers of PwD who use/don't use on the quality of day care. | Mixed Methods | Carers of people with dementia; (N=269). | Daycare | Open-ended survey questions | Content analysis |
| Gilmour, 2002 | New Zealand | To explore family caregivers’ experiences of in-hospital respite care for PwD and the factors that influenced their perceptions of the service. | Qualitative | Carers of people with dementia (4 women caring for their mothers, 2 women caring for husbands, 1 man caring for his mother, 1 man caring for his wife & another caring for his father; (N=9). | Residential respite | Repeated semi-structured interviews | Discourse analysis |
| Gústafsd-óttir, 2014 | Iceland | To explore the longitudinal experiences of families of PwD using specialised day care for the PwD. | Qualitative | Family carers of PwD (4 wives, 2 husbands, 1 daughter and 1 daughter-in-law); (N=8). | Daycare | Repeated semi-structured interviews | Interpretive phenomenological analysis (IPA) |
| Hochgraeber, 2015 | Germany | To understand stakeholders’ perspectives on low-threshold support services (respite) regarding service organisation and conceptualisation. | Qualitative | 3 PwD, 6 family carers, 12 volunteers, 7 coordinators, 3 providers (3 males, 28 females); (N=31). | Daycare, in-home | Semi-structured interviews; Focus groups | Content analysis |
| Holm, 2003 | Australia | To investigate what specific needs the program was intended to meet and how. | Qualitative | Family carers of PwD (N=7). There are indications that staff were also interviewed, but sample size is not stated. | Host-home | Semi-structured interviews | Not clear |
| Huang, 2016 | Singapore | To understand the reasons for non-utilization of day care services for PwD in Singapore using IPA. | Qualitative | Carers of PwD who had never attended day care; (N=16). | Daycare | Semi-structured interviews | IPA |
| Jansen, 2009 | Canada | To explore formal care providers’ perceptions of home and community based services for PwD to inform care quality. | Qualitative | Rural and urban care providers (both professional [e.g. nurses] and non-professional [e.g. care aides]); (N=44). | Daycare, in-home | Semi-structured interviews; Focus groups | Thematic analysis |
| Kirkley, 2011 | UK | To explore the role of organisational culture in barriers & facilitators to person-centred dementia care from PoV of frontline staff and managers in respite. | Qualitative | Strategic managers (34), operational managers (11), frontline staff (17), academics/policy-makers (6 ), independent consultant (2); (N=70) | Daycare, in-home, residential, alternative models e.g. short-break holiday services | Semi-structured interviews; Focus groups | Thematic analysis |
| McGrath, 2000 | Canada | To explore the impact of caring for a family member with Alzheimer’s on the caregiver’s occupational performance and the perceived influence of respite on performance. | Qualitative | Family carers of PwD (2 adult children, 3 spouses); (N=5). | Any 'respite' service | Semi-structured interviews | Qualitative content analysis |
| O'Connell, 2012 | Australia | To examine carers’ experiences and views on using all types of respite. | Mixed Methods | Carers of PwD; (N=62) | Daycare, in-home, residential alternative models e.g. 'regular outings' or 'cottage care' | Open-ended survey questions | Not clear |
| Parahoo, 2002 | UK | To evaluate a domiciliary respite service for carers of younger PwD. | Qualitative | Family carers of PwD (N=8) | In-home | Semi-structured interviews | Not clear |
| Perry, 2001 | Canada | To explore carers experience of a pilot respite program of weekend care for PwD. | Qualitative | Family carers of PwD (N=18) | Weekend respite (overnight service) | Semi-structured interviews | Latent content analysis |
| Phillipson, 2011 (IH*) | Australia | To understand the beliefs that caregivers of PwD have in regard to the use of in-home respite services. | Qualitative | Carers of PwD (10 male, 26 female) (25 spousal , 11 non-spousal); (N=36) | In-home | Semi-structured interviews; Focus groups | Content analysis |
| Phillipson, 2011 (RR*) | Australia | To understand the beliefs that caregivers of PwD have in regard to the use of residential respite. | Qualitative | Carers of PwD (10 male, 26 female) (25 spousal , 11 non-spousal); (N=36) | Residential respite | Semi-structured interviews; Focus groups | Content analysis |
| Phillipson, 2012 | Australia | To understand the beliefs that caregivers of PwD have in regard to the use of daycare. | Qualitative | Carers of PwD (10 male, 26 female) (25 spousal , 11 non-spousal); (N=36) | Daycare | Semi-structured interviews; Focus groups | Content analysis |
| Robinson,2012 | Tasmania | Explores carers perspectives on day care for PwD, including barriers to attendance and strategies to facilitate attendance. | Qualitative | Carers of PwD who had used day care (10) and carers of PwD who have refused day care (17); (N=27) | Daycare | Semi-structured interviews | Analysis conducted "using strategies drawn from grounded theory research" |
| Strang, 2000 | Canada | To understand caregivers’ respite experiences within the context of caring for PwD. | Qualitative | Family carers of PwD (22 spouses, eight adult children and one niece); (N=31) | Any service providing an interval of rest or relief | Repeated semi-structured interviews | Not clear |
| Upton, 2005 | UK | To understand 1) what's it like to be a carer and 2) carers' perceptions and experiences of day and short-term residential/ in-patient respite care. | Qualitative | Spousal caregivers of PwD; (N=46) | Daycare, residential respite | Semi-structured interviews | Not clear |
| Woolrych, 2013 | UK | To understand the experiences of formal carers working within an integrated dementia service (in-home, day care & residential respite). | Qualitative | Formal carers; (N=24) | Integrated daycare, in-home and residential services; semi-private organisation | Semi-structured interviews and focus groups | Thematic analysis |

* Phillipson et al (2011); IH = 2011 paper focused on in-home respite, RR = 2011 paper focused on residential respite
