# Supplementary material for: Key stakeholders’ experiences of respite services for people with dementia and their perspectives on respite service development: a qualitative systematic review
Source: BMC Geriatr. 2017 Dec 7;17:282. doi: 10.1186/s12877-017-0676-0 (PMC5719558; doi:10.1186/s12877-017-0676-0)
Supplement: Supplementary file 3 — Third-Order Interpretations Relating to Key Concepts. (DOCX 16 kb) [file 12877_2017_676_MOESM3_ESM.docx]

**Table 6 - Third-Order Interpretations Relating to Key Concepts**

| **A. Transitioning to Service Use** |
| --- |
| 1. Timely access to services is impeded by cost (carers, providers), a mismatch in service and client perspectives on what constitutes an 'emergency' (carers), and a lack of designated personnel/staff to assist clients in navigating the system (carers, providers) |
| 2. Timely access to services is impeded by under-resourcing and a lack of infrastructure at the systems-level (providers) |
| 3. Service acceptability and fit is initially evaluated by the carer in terms of the services' capacity to 1) recognise and meet the individual needs and preferences of the person with dementia and the carer and 2) to keep the person with dementia safe. (carers) |
| 4. While safe and reliable transport is a key acceptability consideration for clients, and the absence of such a huge access barrier (carers), providers state that it is not always economically feasible to provide this (providers). |
| 5. When service use is unacceptable to the person with dementia, service refusal can cause considerable relational strain in the dyad, which can lead to attrition for those carers who acquiesce, or for whom the conflict makes service use more trouble than its worth (carers) |
| 6. Social and professional (clinicians and HCPs) 'referents' can have a considerable role in normalising and legitimising service use for carer's who hold negative beliefs about services and the associated outcomes of using services. |
| **B. Expanding Organisational Capacity** |
| 1. Increased human and fiscal resources, as well as improvements in infrastructure and governance/guidance are prerequisites for implementing service developments (providers) |
| 2. Service fragmentation and poor infrastructure is considered responsible for poor continuity of care between and across services for people with dementia (providers), however carers feel that discontinuity results when staff don't communicate and co-operate. |
| 3. Both carers and providers acknowledge the importance of the built environment: the preferred environment for carers is 'safe' and 'non-clinical' (carers), while providers need more space and better facilities which serve to offset dementia-related deficits in cognition and functional ability. However, providers maintain they are under-resourced to improve the environment. |
| 4. Providers highlight the need for improved clinical governance, as well as national and international guidance to direct best practice in dementia care (providers) |
| 5. Management often feel ill-equipped to lead on organisational cultural change, particularly with long-term staff that are considered to be set in their attitudes and practices. |
| 6. Staff feel that they cannot implement the improvements desired by clients (i.e. to make services more flexible and responsive to individual needs), because organisational bureaucracy in conjunction with a "risk-averse" managerial culture makes management reluctant to give frontline staff the required autonomy over decision-making. |
| **C. Dementia Care Quality** |
| 1. While carers describe an approach to care that is consistent with the 'person-centred' label (carers), providers often use the label of person-centred care, without implementing its principles in practice (providers). |
| 2. The most valued care component, outside of personal care and medication-monitoring in in-home settings, was meaningful activity for the person with dementia, however carers have divergent views about what constitutes a 'meaningful' activity. |
| 3. Care providers perceive that they are under-resourced to provide dementia care components that are not related to meeting physical care needs, particularly in in-home models of respite, and particularly as the dementia condition progresses. |
| **D. Building a Collaborative Care Partnership** |
| 1. In carer's experiences, adverse outcomes result when services do not listen to their input regarding the person with dementia and their care routines, needs and preferences (carers). |
| 2. Amongst satisfied carers, effective and empathic dyad-service communication, preferably with a designated point of contact, facilitated relationship-building and increased carer's trust in the quality of care (carers). |
| 3. Some providers acknowledge collaborative, and not directive, communication as important, however they also feel that they must be better supported by management to accomplish this (providers). |
| 4. Carers have informational support needs that they feel, if met, would improve the value of the service to them by increasing their care skills and capacity; however they don't always request this information, indicating the importance of the service initiating this conversation with carers (carers). |
| 5. Providers suggest that it is difficult to meet carers informational/educational support needs, as they often arrive at services 'too late' for this type of information to be useful (suggesting an access problem, again systems-level) (providers) |
| **E. Dyad Restoration** |
| 1. For carer's the ultimate aim of using services is to achieve a physical and mental break from caregiving, however their ability to relinquish the carer role is dependent on the carer perceiving that the person with dementia also is benefitting from service use also (carer). |
| 2. The way that carers utilise the time that they gain while the person with dementia is under the care of services influences, as well as the duration of the service use (influenced by service model) substantially impacts their ability to experience restoration (carers) |
| 3. Post-respite, carers continually monitor and evaluate the post-respite experience to determine whether service use is indeed mutually beneficial (outcomes of people with dementia post-respite, stated satisfaction of people with dementia) (carers) - this ongoing evaluation teamed with ongoing open and empathic collaboration with the service, is central to facilitating ongoing dyad restoration through service use. |
